# Posttransplant Allosensitization in Low Immunological Risk Kidney and Kidney-Pancreas Graft Recipients

**DOI:** 10.1155/2014/438945

**Published:** 2014-04-15

**Authors:** Jorge Malheiro, Sandra Tafulo, Leonídio Dias, La Salete Martins, Isabel Fonseca, Manuela Almeida, Sofia Pedroso, Fátima Freitas, Idalina Beirão, António Castro Henriques, António Cabrita

**Affiliations:** ^1^Nephrology and Kidney Transplantation Department, Centro Hospitalar do Porto, Hospital de Santo António, Largo Prof. Abel Salazar, 4099-001 Porto, Portugal; ^2^Centro do Sangue e Transplantação do Porto, Rua de Bolama No. 133, 4200-139 Porto, Portugal

## Abstract

*Introduction*. Posttransplantation allosensitization prevalence and effect on kidney grafts outcomes remain unsettled. *Methods*. Between 2007 and 2012, 408 patients received a primary kidney graft (with 68 patients also receiving a pancreas graft) after a negative cytotoxic crossmatch. All patients had a pretransplant negative anti-HLA screening and 0% panel reactive antibodies. We analyzed retrospectively the results of anti-HLA antibodies screening by Luminex assay, performed between 6 and 24 months after transplant, and searched for the risk factors for antibody positivity and its impact on kidney graft outcomes. *Results*. Anti-HLA antibodies prevalence at 6 months was 17.4%. Previous steroid-insensitive acute rejection was the only risk factor for both anti-HLA classes detected antibodies. Antithymocyte globulin induction was also a risk factor for anti-HLA-I antibodies. Antibody positivity status was associated with reduced graft function at 12 months and graft survival at 5 years (91.5% versus 96.4%, *P* = 0.03). In multivariable Cox analysis, delayed graft function (HR = 6.1, *P* < 0.01), HLA mismatches >3 (HR = 10.2, *P* = 0.03), and antibody positivity for anti-HLA class II (HR = 5.1, *P* = 0.04) or class I/II (HR = 13.8, *P* < 0.01) were independent predictors of graft loss. *Conclusions*. Allosensitization against HLA class II ± I after transplant was associated with adverse kidney graft outcomes. A screening protocol seems advisable within the first year in low immunological risk patients.

## 1. Introduction

Unsensitized kidney and kidney-pancreas recipients may develop* de novo* antibodies against human leukocyte antigens (HLA) after transplantation. Posttransplantation allosensitization prevalence is still disputed, since it depends among other factors on the method used for anti-HLA antibodies detection [1]. For instance, the use of less sensitive techniques such as complement-dependent cytotoxicity (CDC) crossmatch assays in comparison with more sensitive methods as solid-phase assays will result in lower rates of sensitization [2]. This explains that published prevalence of anti-HLA antibodies detected after kidney transplantation ranges from 1.6 to 60% [1]. Uncertainty of the detection of* de novo* anti-HLA antibodies may also result from the use of inaccurate methods to define pretransplant sensitization status. Historical reliance on CDC panel-reactive antibody (PRA) likely missed anti-HLA antibodies present at the time of transplant [3].

Several factors have been associated with the development of* de novo* anti-HLA antibodies such as higher number of HLA mismatches [4, 5], younger recipient age [5], and previous acute rejection episodes [4]. Hourmant et al. [6] showed that previous acute rejection was associated with the development of* de novo* anti-HLA antibodies, donor-specific or not. Besides the clear etiopathogenic connection between anti-HLA antibodies presence and antibody-mediated rejection (AMR), earlier acute cellular rejection (ACR) episodes have also been associated with development of* de novo* anti-HLA antibodies [4, 7].

The deleterious effect of* de novo* anti-HLA antibodies detection on graft outcomes has been demonstrated [1]. A prospective study designed to evaluate the relationship between anti-HLA antibodies development at 1-year after transplant and kidney graft loss showed that antibody-positive recipients had a significantly higher incidence of graft loss after 1-year follow-up [8]. This has led many transplant centers to implement anti-HLA antibodies screening protocols after transplantation, although the target population for these protocols remains matter of discussion [9].

Thus, we decided to analyze in a cohort of low immunological risk patients the relationship between* de novo* anti-HLA antibodies detected at 6-month after transplant and kidney graft outcomes. Accordingly, we selected for analysis only patients without allosensitization before transplant as determined by CDC PRA and/or a screening by Luminex solid-phase assay. An association between anti-HLA antibodies detection and significant graft outcomes would support the clinical usefulness of this screening strategy in low risk patients.

## 2. Material and Methods

### 2.1. Subjects

We retrospectively analyzed 579 adult patients who received a first kidney (*n* = 498) or a kidney-pancreas (*n* = 81) transplant between 2007 and 2012, with a functioning kidney graft for at least 6 months, and in whom a CDC PRA test and anti-HLA antibodies screening had been performed before transplant. All antibody-positive patients underwent LABScreen test for detection of anti-HLA antibodies around the 6th month after transplant. Antibody-negative patients were selected if they had a negative screening performed between the 6th and the 24th month following transplant; in patients with multiple screenings only those with negative results in all of them were selected. We used stringent criteria to select patients without pretransplant allosensitization in order to analyze its prevalence and effect after transplantation. Hence, we considered only primary graft recipients and we excluded patients with a pretransplant (historical or current) CDC PRA > 0% and/or a positive anti-HLA antibodies screening (*n* = 161) and patients with positive screening posttransplant after a negative one at 6 months (*n* = 10), defining the remaining 408 patients as the study population. All patients were transplanted with a negative pretransplant T- and B-lymphocyte cytotoxicity crossmatch.

The Institutional Review Board at Centro Hospitalar do Porto approved this study.

### 2.2. Anti-HLA Screening and % PRA

CDC PRA test was performed before transplant in all patients with sera collected every 3 months while in waiting list, using total peripheral blood lymphocytes collected from a HLA-typed representative donor population. It was considered positive if cell lyses remained present after dithiothreitol (DTT) treatment, identifying only IgG anti-HLA isotypes positive cases.

Pre- and posttransplant anti-HLA IgG antibodies were tested by multiplex microsphere based flow cytometry (Luminex Technology, LABScreen Mixed kit, OneLambda, Canoga Park, CA). Color-coded microspheres, coated with the major HLA class I and II antigens, were incubated with the serum for 30 min at room temperature in the dark. After three washes the samples were incubated with 100 *μ*L of 1 : 100 phycoerythrin-conjugated goat antihuman IgG (One Lambda Inc.). Finally, after two washes, the fluorescent signal intensity for each microsphere was measured using LABScan 100 Flow analyzer (One Lambda Inc.). The cutoff for positive samples was the normalized background (NBG) ratio recommended by the manufacturer and performed by the HLA fusion software (One Lambda).

In deceased donor recipients, no flow cytometry crossmatch was performed since patients had a CDC PRA = 0% and no detectable anti-HLA antibodies before transplant. Flow cytometric crossmatch was performed in living donor recipients as standard practice (all transplants were carried out with a negative T- and B-cell flow crossmatch).

### 2.3. Pretransplant Induction Protocol and Maintenance Immunosuppression

Induction therapy was used in 370 patients (90.7%), with 256 patients receiving an anti-IL-2 receptor monoclonal antibody (basiliximab, 20 mg twice at day 0 and 4) and 114 patients receiving polyclonal antithymocyte globulin (ATG Fresenius, 3 mg/kg for 5–7 days). Per protocol, kidney-pancreas recipients (*n* = 68) received ATG for induction, with only 4 patients receiving basiliximab instead. ATG was used in kidney-only recipients at the clinician discretion, mainly due to a high number of HLA mismatches. All enrolled recipients had similar triple maintenance immunosuppression, consisting of oral tacrolimus (FK-506), mycophenolate mofetil (MMF), and methylprednisolone (MP)/prednisolone. FK-506 was started at the dose of 0.1–0.15 mg/kg/day, and the dose was adjusted to maintain a trough level of FK-506 in whole blood between 8 and 12 ng/mL during the first month postoperatively, between 7 and 10 ng/mL during 2-3 months after transplant and between 5 and 8 ng/mL thereafter. MMF was started at the dose of 2000 mg/day, with the dose decreasing to 1000–1500 mg/day during the first month postoperatively, depending on white blood cells count. Methylprednisolone was administered intravenously at doses of 500, 250, and 125 mg/day on the day of transplantation, on day 1-2 and day 3-4 after the operation, respectively. Oral prednisolone was started on day 5 after the operation at the dose of 20 mg, being then tapered to 5–10 mg/day within 2-3 months after transplant. Living donor recipients (*n* = 76) were prescribed FK-506 and MMF 7 days before transplant.

### 2.4. Data Analysis and Outcomes

Data regarding recipient and donor characteristics and pre- and posttransplantation variables were collected retrospectively in all patients. Delayed graft function was defined as dialysis requirement in the first week after transplant. Estimated glomerular filtration rate (eGFR) was evaluated at 12 months after transplant in patients with a functioning graft at that moment (*n* = 404), using the 2006 MDRD equation. Graft survival was analyzed considering graft failure censored for death with a functioning graft.

### 2.5. Rejection Diagnosis and Treatment

Allograft rejection was defined as biopsy proven rejection (specimens were evaluated by light microscopy and immunofluorescence staining for C4d) and classified according to Banff classification as updated in 2007. Mild acute cellular rejection (ACR Banff grade I) was treated with pulse steroids (500 mg MP for 3 days) and increased maintenance immunosuppression. All other ACR episodes were treated with ATG. Antibody-mediated rejection (AMR) was also treated with pulse steroids and intravenous immunoglobulin 2 g/kg (maximum 140 g) divided in two doses associated with plasmapheresis (at least 3–5 sessions). Acute rejection episodes were further classified as steroid-sensitive rejections (ACR Banff grade I) or steroid-insensitive rejections (ACR Banff grade II and III and AMR).

### 2.6. Statistical Analysis

Continuous data was described using mean (standard deviation) or median (interquartile range) and categorical data was expressed as number (frequencies). Demographic, clinical, and immunological features and posttransplant anti-HLA antibodies status were compared using Pearson chi-square test or Fisher's exact test for categorical data and Student's *t*-test or Mann-Whitney *U* test for continuous data, as appropriate. Logistic regression analysis was used to determine significant associations between studied variables and 6-month presence of anti-HLA antibodies, using a multivariable model that included variables presenting *P* ≤ 0.1 in univariable analysis (ATG use, time on dialysis, kidney-pancreas graft, acute rejection type, recipient age, donor age, and ABDR mismatches) (data not shown). Graft survival curves were visualized using Kaplan-Meier method, with in-between groups comparison done by log-rank test. Univariable and multivariable Cox proportional hazards analysis was applied to assess independent predictors of censored graft failure; a multivariable model (including variables presenting *P* ≤ 0.1 in univariable analysis: ATG use, recipient age, donor age, delayed graft function, ABDR mismatches, and anti-HLA antibodies screen) was constructed to adjust for potential confounders.

A two-sided *P* value < 0.05 was considered as statistically significant. Statistical calculations were performed using SPSS for Mac, version 20.0 (SPSS Inc., Chicago, IL, USA).

## 3. Results

In our cohort of 408 unsensitized kidney and kidney-pancreas recipients, anti-HLA antibodies were detected at 6-month after transplant in 71 patients (17.4%), with 49 (12.0%) being positive for anti-HLA class I, 12 (2.9%) for anti-HLA class II, and 10 (2.5%) for anti-HLA class I and II antibodies. Median follow-up was 44 months (interquartile range: 31–60).

### 3.1. Baseline Characteristics and Variables Associated with the Presence of Anti-HLA Antibodies (Table 1)

Patients with detectable anti-HLA antibodies were significantly younger, had a younger donor, and were predominantly kidney-pancreas recipients. They had a significant higher mean HLA mismatches and underwent induction immunosuppression with ATG more frequently. At 6 months after transplant, occurrence of previous acute rejection was more common in patients with detectable anti-HLA antibodies. No significant difference was found in delayed graft function prevalence.

A multivariable logistic regression analysis was performed to determine risk factors for anti-HLA antibodies positivity at 6 months (Table 2). Steroid-insensitive acute rejection episodes were a potent risk factor (OR = 6.47, *P* < 0.01) for anti-HLA antibodies presence of any class. Steroid-sensitive acute rejection episodes were marginally associated (OR = 3.90, *P* = 0.05) with anti-HLA class II detection. Remarkably, ATG induction was a risk factor (OR = 4.04, *P* < 0.01) for anti-HLA class I detection.

### 3.2. Acute Rejection Characteristics and Anti-HLA Antibodies Detection

Forty-four patients had acute rejection in the first 6 months after transplant, with 22 rejections being classified as ACR grade I, 17 as ACR grade II, and 5 as AMR (2 of them had also ACR grade I and 1 had also ACR grade II). Previous AMR and ACR grade II episodes were more frequent in patients with detectable anti-HLA antibodies than in those without them [AMR: 4 (5.6%) versus 1 (0.3%) *P* < 0.01; ACR grade II: 6 (8.5%) versus 11 (3.3%) *P* = 0.047]. Differently, ACR grade I occurrence was similar between groups [5 (7.0%) versus 17 (5.0%), *P* = 0.50]. No significant difference was detected in acute rejection between patients with and without ATG induction (7.9% versus 11.9%, resp., *P* = 0.24).

### 3.3. Anti-HLA Antibodies and Kidney Graft Outcomes after Antibody Testing

At 12 months after transplant, antibody-positive recipients had a significantly lower mean eGFR than antibody-negative patients (48.5 ± 20.1 and 54.0 ± 18.1 mL/min, resp., *P* = 0.04).

**Figure 1:**
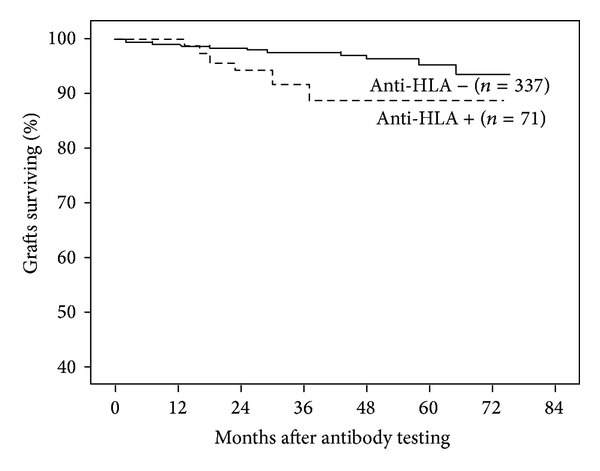
Graft survival censored for death with a functioning graft according to anti-HLA antibodies presence at 6 months after transplant. Antibody-positive patients (*n* = 71) showed a significantly lower survival rate at 5 years than antibody-negative patients (*n* = 337) (91.5% versus 96.4%, resp., log-rank test *P* = 0.03).

**Figure 2:**
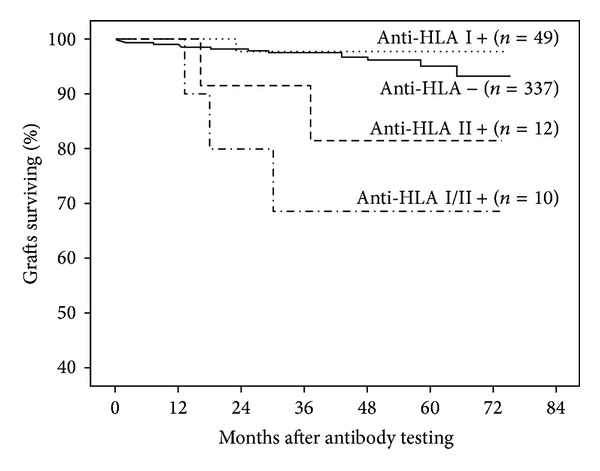
Graft survival censored for death with a functioning graft according to presence of anti-HLA antibodies against class I, class II, or class I/II at 6 months after transplant. Patients with antibody positivity for class II (*n* = 12) (83.3%, log-rank test *P* = 0.02) or class I/II (*n* = 10) (70%, log-rank test *P* < 0.01) showed a significantly lower survival rate at 5 years than antibody-negative patients (*n* = 337). Graft survival was similar in antibody-negative patients and those positive only for class I (*n* = 49) anti-HLA antibodies (96.4% versus 98% resp., log-rank test *P* = 0.76).

After 5-year follow-up, 6 (8.5%) antibody-positive recipients lost their grafts, while this occurred in only 12 (3.6%) of antibody-negative patients (Figure 1). When we analyzed antibody positivity accordingly to HLA class, censored graft loss was associated with the presence of anti-HLA class II (2 patients lost their graft) or anti-HLA class I/II (3 patients lost their graft), but not with the presence of anti-HLA class I (1 patient lost his graft) antibodies only (Figure 2). Of the 18 patients with graft failure, graft biopsies obtained between antibody testing and failure were available in 10 patients (8 from antibody-positive and 2 from antibody-negative patients). In antibody-positive patients, 6 presented a grade II/III and 2 patients a grade I interstitial fibrosis and tubular atrophy according to Banff'07 classification; simultaneously 5 patients had signs of chronic active antibody-mediated rejection without C4d deposition (peritubular capillaries and/or glomerular inflammation) and 4 patients presented with mild to moderate interstitial infiltration. In antibody-negative patients, 1 biopsy showed a grade I interstitial fibrosis and tubular atrophy together with mild interstitial infiltration; the other specimen presented no significant changes.

Kidney graft loss occurred in 16 (4.7%) kidney-only and in 2 (2.9%) kidney-pancreas graft recipients (*P* = 0.75). Given the association of ATG induction with anti-HLA class I positivity, we analyzed graft survival considering induction therapy used. Most graft losses occurred in patients without induction therapy or induced with basiliximab (*n* = 16). In patients induced with ATG (*n* = 114) only 2 lost their grafts (none from the antibody-positive group).

Furthermore, a Cox proportional hazard model was constructed to explore predictors of censored graft loss (Table 3). In the multivariable analysis, anti-HLA class II or anti-HLA class I/II antibodies positivity, delayed graft function and HLA ABDR mismatches > 3 were significant predictors of censored graft loss.

### 3.4. Patient Deaths after Antibody Testing

Six patients died during follow-up, 5 (death causes: neoplasia 2, cardiovascular disease 2, and septicemia 1) in the antibody-negative group and 1 (from septicemia) in the antibody-positive group (*P* = 1.0).

## 4. Discussion

In our cohort of kidney and kidney-pancreas recipients without pretransplant allosensitization, anti-HLA antibodies positive screening at 6 months was associated with worse kidney graft function and survival. Prevalence of* de novo* anti-HLA antibodies was 17.4%, being anti-HLA class I in 69%, anti-HLA class II in 17%, and against both classes in 14% of antibody-positive patients. Similar results were reported in kidney and kidney-pancreas recipients in whom alloantibody analysis was performed by solid-phase assays. In one study [7] of 277 patients (77% kidney and 23% kidney-pancreas recipients), 21.8% of those without allosensitization before transplantation became allosensitized at a mean 2.6 years after transplant, against HLA class II in the majority of cases. Forty patients from a cohort of 167 pancreas graft recipients with 91% receiving simultaneously a kidney graft (7 patients were HLA-sensitized before transplant) had a positive screening for anti-HLA antibodies at a median follow-up of 1-year, with detected antibodies equally distributed between HLA classes (47.5% for each, with 5% positive for both) [10]. Our cohort presented a lower prevalence of HLA-sensitization, probably due to its low immunological risk character and an earlier done antibody screening.

We acknowledge that anti-HLA class I antibodies high prevalence in our population is in contrast with published data in which anti-HLA class II predominates within* de novo* anti-HLA antibodies [4, 5, 11], but most of these studies analyzed only donor-specific anti-HLA antibodies prevalence, without mention to overall anti-HLA antibodies prevalence and had a longer median follow-up to antibody detection (at least 17 months). Additionally, we found an independent association between induction therapy with ATG and positivity for anti-HLA class I antibodies at 6 months, also reported by others authors [6, 12]. This association cannot be attributed solely to a more frequent use of ATG induction in recipients with a higher immunological risk, since it remained significant in a multivariable regression model that included variables related with allosensitization risk (HLA mismatches, kidney-pancreas transplantation). In a case control study [12] that included two groups of patients well matched for immunological risk variables but differing in the use of ATG induction, a significant increase (over 10%) of anti-HLA class I antibodies after transplant, using a FlowPRA solid-phase assay, was observed more frequently in ATG induced patients than in those without induction therapy (22.2% versus 0%; *P* = 0.02). Hourmant et al. [6], reporting in a population of 1229 kidney graft recipients (10% also received a pancreas graft), found a prevalence of 16.8% anti-HLA antibodies at a mean follow-up of around 6 years using a ELISA screening test. Antibody-positive patients had received more frequently induction with ATG (76% versus 58%; *P* < 0.01). No information is given about against which class (I and/or II) were the detected anti-HLA antibodies. The main issue here is if this association results from a laboratory interference of a xeno-antibody present in ATG preparation [13] with no foreseeable effect on the graft or if it results from an imbalance between T- and B-cell populations with a stronger depletion effect in the former (including regulatory T-cells) allowing for humoral responses to evolve [14]. Our results are in accordance with the first proposition, since we reported only 2 graft losses in the ATG induced group and no significant difference was detected in acute rejection between patients with and without ATG induction. High frequency of positivity for anti-HLA class I antibodies in our cohort is probably associated with the precociousness of our antibody screening in relation to ATG use, not only as induction therapy but also in the treatment of the steroid-insensitive acute rejections episodes (*n* = 22).

Acute rejection episodes, if classified as vascular ACR or AMR (steroid-insensitive), were a strong risk factor for anti-HLA antibodies detection, independently of HLA class. In kidney-pancreas recipients, it has been shown that vascular (Banff grade II or III) kidney graft ACR was significantly more common in patients with posttransplant detection of anti-HLA antibodies [10]. Recently, a retrospective study in 2079 kidney-only graft recipients recognized that cases of vascular ACR should be reevaluated if simultaneous presence of donor-specific anti-HLA antibodies was detected [15]. With their reevaluation, more than a half of the cases formerly classified as vascular ACR would be reclassified as vascular AMR, an entity that presented particularly poor graft outcomes. An association of ACR and later development of* de novo* anti-HLA antibodies may relate with the degree of microcirculatory inflammation present at the time of the ACR, in particular the sensitizing effect of upregulated HLA proteins expression in the peritubular capillaries [16]. Moreover, histopathological analysis of vascular rejection biopsies showed that concomitant presence of peritubular capillaritis was very common (around 90%) [17]. We found no significant association between the number of HLA mismatches and appearance of anti-HLA antibodies in the multivariable logistic analysis, in spite of a significant higher HLA mismatches mean in antibody-positive recipients. Lachmann et al. [18] found no significant difference between ABDR mismatches mean and posttransplant antibody screening status. Others [4, 19] found HLA mismatches number to be associated with* de novo* donor-specific anti-HLA antibodies, particularly for HLA-DR mismatches.

The lack of information regarding specificities of the detected antibodies, namely the recognition of donor-specific antibodies, represents a limitation of this study. Nevertheless, we found that anti-HLA antibody detection was a significant predictor of kidney graft outcome, as it was associated with lower eGFR at 1-year posttransplant and with reduced censored graft survival at 5-year follow-up. When anti-HLA antibodies class was considered, we detected that only antibodies against class II or class I/II were significant predictors of censored graft failure. A multivariable Cox analysis confirmed these results, besides identifying delayed graft function and a higher number of HLA mismatches as other predictors of graft failure. Several studies after 2000, using the solid-phase assays, have demonstrated anti-HLA antibodies presence to be a significant predictor of poorer graft survival [6, 20].* De novo* posttransplant detection of alloantibodies in kidney and kidney-pancreas recipients was associated with a significantly lower death censored graft survival [7]. More recently, with the development of single antigen beads technology the emphasis has been put in the role of* de novo* donor-specific anti-HLA antibodies. Several studies show that anti-HLA antibodies detrimental effect on graft survival is restricted to* de novo* donor-specific antibodies [4, 21, 22]. Interestingly, it has been shown in a cohort of kidney-pancreas patients that the significant detrimental impact of anti-HLA antibodies on kidney graft outcome (death-censored graft survival at 9-years was 53% in anti-HLA positive and 89% in anti-HLA negative patients) was due to donor-specific antibodies, since patients with anti-HLA antibodies non-donor-specific and those anti-HLA negative had similar graft survival (92% versus 89%) [10]. Additionally, they showed that although anti-HLA antibodies were equally distributed between HLA classes, when they analyzed antibody-positive patients, with or without donor-specific antibodies, antibodies anti-HLA class II predominated in the former and anti-HLA class I in the latter. Nonetheless, others have demonstrated poorer graft survival associated with anti-HLA antibodies irrespective of donor specificity [6, 23]. Colleagues from Charité Hospital in Berlim [18] screened kidney graft recipients (11% also received a pancreas) at a median of 5 years after transplant with Luminex solid-phase assays for anti-HLA antibodies presence. Patients with detectable anti-HLA antibodies had significantly worse graft survival than those that remained unsensitized (63% versus 83%) at 5.5-year follow-up. When kidney graft survival analysis was stratified for anti-HLA antibodies presence and their specificities, they found that both donor-specific (49%) and non-donor-specific antibodies (70%) were associated with worse graft survival than antibody-negative patients (83%). They also reported that eGFR at time of antibody testing was significantly lower in antibody-positive patients.

Graft histopathological data from available biopsies of patients with eventual censored graft failure displayed mild to moderate chronic damage pattern, frequently associated with presence of inflammatory lesions, recognized as both cellular and antibody-mediated. All graft failures in antibody-positive patients occurred at least 1 year after antibody testing, further underlining the indolent nature of the processes at play. An independent detrimental effect on kidney graft survival (through loss by chronic rejection) of anti-HLA antibodies development within 1 year after transplantation has been reported [24]. Campos et al. [25] showed that the presence of anti-HLA class II antibodies (alone or concomitant with class I) was a predictor of graft loss due to chronic allograft nephropathy, independently from the degree of renal function decline already observed at the time of antibody testing.

The definition of our cohort as low immunological risk may be criticized given the inclusion of kidney-pancreas patients, although we have shown that double transplantation was not an independent risk factor for antibody positivity nor was it associated with higher graft loss. The different timing for the evaluation of anti-HLA antibodies (at 6 months in antibody-positive and between 6 and 24 months after transplant in antibody-negative patients) may be disputed. However, we believe that the exclusion of every patient with a positive screening after the 6-month screening minimizes it.

Our results about the relevance of anti-HLA antibodies screening at 6 months after transplant should be carefully assessed. We describe a probable laboratorial interference with the detection of anti-class I HLA antibodies associated with ATG use that renders those results clinically irrelevant. We were unable to determine the precise time interval between ATG use and antibody testing. Probably, a screening done at 12 months after transplant would allow us to surpass it. Nevertheless, a significant association between a positive screening for anti-class II±I HLA antibodies and kidney graft adverse outcomes was found. Naturally, a positive screening should prompt clinicians to perform an assay for identification of antibody specificities in order to define eventual donor specificity.

## 5. Conclusions

We recommend caution in the interpretation of positive screening for anti-HLA antibodies against class I in patients that recently received ATG therapy. Nonetheless, our results show that anti-HLA antibodies screening after transplant should be a tool in the clinical management of patients with low immunological risk. It is a first step in the study of allosensitization, identifying those in need of more accurate but also more expensive assays, thus allowing for a more adequate allocation of means. A screening protocol for detection of* de novo* allosensitization within the first year after transplant seems advisable for most transplanted patients.

## Figures and Tables

**Table 1:** Baseline characteristics and events at 6-months posttransplant for all patients and between anti-HLA (−) and anti-HLA (+) antibodies groups.

Variables	All patients *N* = 408	anti-HLA (−) *N* = 337	anti-HLA (+) *N* = 71	*P* value
Recipient variables				
Age (years), mean ± SD	43.5 ± 15.2	44.4 ± 14.7	39.1 ± 17.2	0.02
Female gender, *n* (%)	133 (32.6%)	105 (31.2%)	28 (39.4%)	0.18
Time on dialysis (mo), median (IQR)	41.0 (17.4–74.3)	41.8 (17.9–75.1)	28.4 (10.9–53.8)	0.06
Previous blood transfusions, *n* (%)	130 (31.9%)	107 (31.8%)	23 (32.4%)	0.92
Females with previous pregnancies, *n* (%)	56 (42.1%)	47 (44.8%)	9 (32.1%)	0.29
KP recipients, *n* (%)	68 (16.7%)	50 (14.8%)	18 (25.4%)	0.03
Donor variables				
Age (years), mean ± SD	44.5 ± 15.6	45.8 ± 14.7	38.3 ± 18.3	<0.01
Female gender, *n* (%)	143 (35.0%)	117 (34.7%)	26 (36.6%)	0.82
Living donor, *n* (%)	76 (18.6%)	65 (19.3%)	11 (15.5%)	0.46
Transplantation variables				
ABDR HLA mismatches, mean ± SD	3.89 ± 1.41	3.82 ± 1.43	4.21 ± 1.25	0.02
ABDR mismatches >3, *n* (%)	261 (64.0%)	211 (62.6%)	50 (70.4%)	0.21
Cold ischemia time (h), median (IQR)	14.7 (10–18.2)	14 (9.5–18)	16.5 (11–19)	0.91
Induction therapy				
ATG use, *n* (%)	114 (27.9%)	81 (24.0%)	33 (46.5%)	<0.01
Basiliximab use, *n* (%)	256 (62.7%)	222 (65.9%)	34 (48.6%)	0.01
No induction, *n* (%)	38 (9.3%)	34 (10.1%)	4 (5.6%)	0.37
Posttransplant events at 6-mo				
Blood transfusions, *n* (%)	29 (7.1%)	22 (6.5%)	7 (9.9%)	0.31
Delayed graft function, *n* (%)	73 (17.9%)	58 (17.2%)	15 (21.1%)	0.43
Acute rejection, *n* (%)	44 (10.8%)	29 (8.6%)	15 (21.1%)	<0.01
Steroid-sensitive, *n* (%)	22 (5.4%)	17 (5.0%)	5 (7.0%)	0.50
Steroid-insensitive, *n* (%)	22 (5.4%)	12 (3.5%)	10 (14.1%)	<0.01

HLA: human leukocyte antigen; SD: standard deviation; mo: months; IQR: interquartile range; KP: kidney-pancreas; h: hours; ATG: anti-thymocyte globulin.

**Table 2:** Risk factors for anti-HLA antibodies positivity at 6 months by multivariable* logistic regression analysis.

	Odds ratio	95% IC	*P* value
Risk factors for anti-HLA (+)			
ATG use	3.05	1.49–6.25	<0.01
Acute rejection			
No episode	Reference		
Steroid-sensitive	1.75	0.59–5.16	0.31
Steroid-insensitive	6.47	2.55–16.42	<0.01
Risk factors for anti-HLA class I (+)			
ATG use	4.04	1.89–8.65	<0.01
Acute rejection			
No episode	Reference		
Steroid-sensitive	1.05	0.29–3.87	0.94
Steroid-insensitive	4.45	1.63–12.09	<0.01
Risk factor for anti-HLA class II (+)			
Acute rejection			
No episode	Reference		
Steroid-sensitive	3.90	0.99–15.42	0.05
Steroid-insensitive	5.05	1.46–17.46	0.01

*Variables included in the model are ATG use, time on dialysis, kidney-pancreas graft, acute rejection type, recipient age, and donor age, ABDR mismatches.

HLA: human leukocyte antigen; IC: confidence interval; ATG: antithymocyte globulin.

**Table 3:** Predictors of censored kidney graft loss by Cox proportional hazard analysis.

	Hazard ratio	95% IC	*P*-value
Univariable analysis			
Recipient age	1.05	1.01–1.08	0.01
Male (versus female) recipient	0.58	0.23–1.46	0.26
Living (versus deceased) donor	0.04	0.01–5.87	0.20
Donor age	1.03	0.99–1.07	0.07
Male (versus female) donor	0.48	0.19–1.25	0.13
Delayed graft function	5.85	2.30–14.85	<0.01
ATG use	0.30	0.07–1.29	0.10
ABDR HLA mismatches >3	9.39	1.25–70.60	0.03
Time on dialysis ≥48 (versus <48) mo	2.05	0.79–5.28	0.14
Acute rejection			
No episode	Reference		
Steroid-sensitive	1.17	0.15–8.83	0.88
Steroid-insensitive	2.02	0.46–8.86	0.35
Kidney-pancreas recipient	0.62	0.14–2.72	0.53
Anti-HLA antibodies at 6 mo after transplant			
Negative	Reference		
Class I	0.71	0.09–5.46	0.74
Class II	4.79	1.07–21.40	0.04
Class I + II	10.95	3.08–38.96	<0.01
Multivariable analysis*			
Recipient age	1.02	0.98–1.07	0.29
Donor age	1.02	0.98–1.06	0.45
ATG use	0.44	0.09–2.06	0.30
Delayed graft function	6.11	2.21–16.92	<0.01
ABDR HLA mismatches >3	10.17	1.32–78.55	0.03
Anti-HLA antibodies at 6 mo after transplant			
Negative	Reference		
Class I	1.23	0.15–10.11	0.85
Class II	5.12	1.07–24.53	0.04
Class I + II	13.79	3.41–55.77	<0.01

*Variables included in the model are ATG use, recipient age, donor age, delayed graft function, ABDR mismatches, and anti-HLA antibodies screen.

IC: confidence interval; ATG: antithymocyte globulin; mo: months; HLA: human leukocyte antigen.
